# Quantifying HER-2 expression on circulating tumor cells by ACCEPT

**DOI:** 10.1371/journal.pone.0186562

**Published:** 2017-10-30

**Authors:** Leonie Zeune, Guus van Dalum, Charles Decraene, Charlotte Proudhon, Tanja Fehm, Hans Neubauer, Brigitte Rack, Marianna Alunni-Fabbroni, Leon W. M. M. Terstappen, Stephan A. van Gils, Christoph Brune

**Affiliations:** 1 Department of Applied Mathematics, MIRA Institute, University of Twente, Enschede, The Netherlands; 2 Department of Medical Cell BioPhysics, MIRA Institute, University of Twente, Enschede, The Netherlands; 3 Circulating Tumor Biomarkers Laboratory, SiRIC, Translational Research Department, Institut Curie, PSL Research University, Paris, France; 4 CNRS UMR144, Institut Curie, PSL Research University, Paris, France; 5 Department of Gynecology and Obstetrics, Heinrich-Heine-University, Duesseldorf, Germany; 6 Department of Gynecology and Obstetrics, Ludwig-Maximilians-University, Munich, Germany; The Ohio State University, UNITED STATES

## Abstract

Circulating tumor cells (CTCs) isolated from blood can be probed for the expression of treatment targets. Immunofluorescence is often used for both the enumeration of CTC and the determination of protein expression levels related to treatment targets. Accurate and reproducible assessment of such treatment target expression levels is essential for their use in the clinic. To enable this, an open source image analysis program named ACCEPT was developed in the EU-FP7 CTCTrap and CANCER-ID programs. Here its application is shown on a retrospective cohort of 132 metastatic breast cancer patients from which blood samples were processed by CellSearch^®^ and stained for HER-2 expression as additional marker. Images were digitally stored and reviewers identified a total of 4084 CTCs. CTC’s HER-2 expression was determined in the thumbnail images by ACCEPT. 150 of these images were selected and sent to six independent investigators to score the HER-2 expression with and without ACCEPT. Concordance rate of the operators’ scoring results for HER-2 on CTCs was 30% and could be increased using the ACCEPT tool to 51%. Automated assessment of HER-2 expression by ACCEPT on 4084 CTCs of 132 patients showed 8 (6.1%) patients with all CTCs expressing HER-2, 14 (10.6%) patients with no CTC expressing HER-2 and 110 (83.3%) patients with CTCs showing a varying HER-2 expression level. In total 1576 CTCs were determined HER-2 positive. We conclude that the use of image analysis enables a more reproducible quantification of treatment targets on CTCs and leads the way to fully automated and reproducible approaches.

## Introduction

Peripheral blood tumor load represented by CTC is associated with poor outcome in cancer patients [1–5]. The availability of CTCs allows for the assessment of treatment targets and opens the avenue to provide CTC-based therapy to the patient. The ability to detect treatment targets on CTC has been demonstrated in a variety of studies [6–12]. Before this information can be used in the clinic it is imperative that such a target can be reproducibly and consistently quantified on the CTC at different clinical sites. Although the majority of multicenter studies have been performed with the FDA cleared CellSearch^®^ system, in recent years many systems have been introduced to detect and isolate CTCs [13,14,15]. The lack of a unified approach to designate a cell as a CTC, and to determine whether or not a CTC expresses a treatment target, leads to large differences in reported CTC numbers and positivity rates for potential therapeutic targets such as HER-2 between various studies urging the need for standardization. To address this issue a CTC image analysis algorithm for identification and characterization of CTC is being developed in the EU funded CANCER-ID & CTCTrap programs. Here we introduce the first version of the Open Source program named ACCEPT (**A**utomated **C**TC **C**lassification **E**numeration and **P**heno**T**yping) that allows for the quantification of treatment targets on annotated CTCs. ACCEPT is a toolbox in which a novel efficient parameter-free multi-scale segmentation method is used to identify objects in images captured by several CTC platforms [16]. Here we investigate a retrospective set of images generated from CTCs detected in metastatic breast cancer patients’ blood by the CellSearch^®^ system in which the expression of HER-2 is assessed. Images generated by the CellSearch^®^ system were used as this system is standardized and in use in multiple centers. But any annotated set of tiff images can be analyzed using the toolbox, allowing for the quantification of markers independent of the CTC enrichment platform that is used. The toolbox is available for use at https://github.com/LeonieZ/ACCEPT.

## Materials and methods

### CTC enumeration and HER-2 assessment

The CellSearch^®^ system (Menarini Silicon Biosystems Inc, Huntingdon Valley, PA, USA) was used to enumerate CTCs and to assess their relative HER-2 expression. The cells enriched from 7.5 ml of blood by EpCAM-expression were labeled with phycoerythrin (PE) conjugated antibodies directed against epithelial cell specific cytokeratins (CKs), with an allophycocyanin conjugated (APC) antibody directed against leukocyte specific CD45, and with a Fluorescein (FITC) conjugated antibody (HER81) directed against HER-2. Additionally, cells were stained with the nuclear dye 4,6-diamidino-2-phenylindole (DAPI) to identify the nucleus. The CellTracks Analyzer II^®^ (Menarini Silicon Biosystems Inc) was used to acquire digital images of the four different fluorescent dyes using a 12-bit camera that are transformed and store as 8-bit images during archiving. Trained operators reviewed thumbnail images generated by the CellTracks Analyzer II^®^ to count and determine the HER-2 expression of the CTCs according to the manufacturer’s instructions.

### Cell lines

Breast carcinoma cell lines SKBR-3, MDA-MB 453 and MDA-MB 231 were obtained from ATCC (Manassa, VA, USA) and cultured in DMEM (Gibco, Life Technologies, Waltham, MA, USA) containing 2 mM L-glutamine (G7513, Sigma-Aldrich), 100 U/mL penicillin and 100 μg/mL streptomycin (P4333, Sigma-Aldrich) and 10% FBS (F4135, Sigma-Aldrich) at 37°C in a humidified 5% CO_2_ incubator. Cells were trypsinized at about 80% confluence with 0.05% Trypsin-EDTA (1X) with Phenol Red (Gibco, Life Technologies). HER-2 quantification was performed with flow cytometry using the QuantiBRITE^®^ PE quantification kit (BD biosciences, San Jose, California) using a previously published protocol [17]. Peripheral blood of healthy donors was spiked with either 1500 SKBR-3, 500 MDA-MB 453 or 500 MDA-MB 231 cells and processed with the CellSearch^®^ system.

### Patients

In this study CTC images generated by the CellSearch^®^ system (Menarini Silicon Biosystems Inc) from 132 patients with metastatic breast cancer were used. 80 patients (36 recruited in the Department of Gynecology and Obstetrics—Ludwig-Maximilians-University, Munich and 44 in the Department of Gynecology and Obstetrics—Heinrich-Heine-University, Duesseldorf) were enrolled in the Detect III study (NCT01619111). The other 52 patients were enrolled in the BEVERLY02 study from Paris (NCT00717405). All patient identifying information is maintained at the clinical sites and no access to this information was available for our analysis. Approval for HER-2 CTC analysis was obtained by the independent ethics committee for both, the Detect III study (Ethical Committee of the Heinrich-Heine University Duesseldorf) and the BEVERLY02 study (Comité de Protection des Personnes Sud Méditerranée I), and all patients provided written informed consent.

### Image analysis

The images were reanalyzed using the newly developed image analysis toolbox for CTC analysis ACCEPT (**A**utomated **C**TC **C**lassification **E**numeration and **P**heno**T**yping). The software is written in Matlab 2016a (Mathworks, Natick, MA). Next to the source code a compiled standalone executable version, that includes the MATLAB Runtime and allows royalty-free deployment for users who do not need MATLAB, is also available. In ACCEPT objects of interest are segmented based on shape contours and intensity. The underlying algorithm to automatically detect multiple objects with different scales is described in detail elsewhere [16,18,19]. The main tool of the ACCEPT toolbox is a fully automated detection and classification approach for blood samples but in this paper we introduce a second tool, called the Marker Characterization tool, that reproducibly evaluates the marker expression of prescored cells and can aid users to manually score or quantify cells and their fluorescent signals.

The thumbnail images of all 4084 manually scored CTCs out of 132 patients were loaded into the ACCEPT algorithm. The images that are stored as 8-bit images by the CellSearch^®^ system with an image brightness automatically adjusted relative to the brightest pixel in the channel, are rescaled before processing to their true intensity values by extracting the original minimum and maximum value in each channel from the accompanying tiff header. Afterwards the outline of all events present in these images was automatically detected in each channel. Based on these contours, we extracted seven different measurements per object and per fluorescent channel, i.e. eccentricity (circularity measure), perimeter, mean intensity, maximum intensity, size, standard deviation of the intensity, mass (sum of all intensity values) and perimeter2area (circularity measure). Moreover, we extracted the relative overlay of the signals in the DAPI and CD45 channels and in the DAPI and CK channels. The thumbnail images together with the extracted contour and measurements are presented to the operator to determine the HER-2 expression.

## Results

### Visualization of CTC by ACCEPT

**Fig 1:**
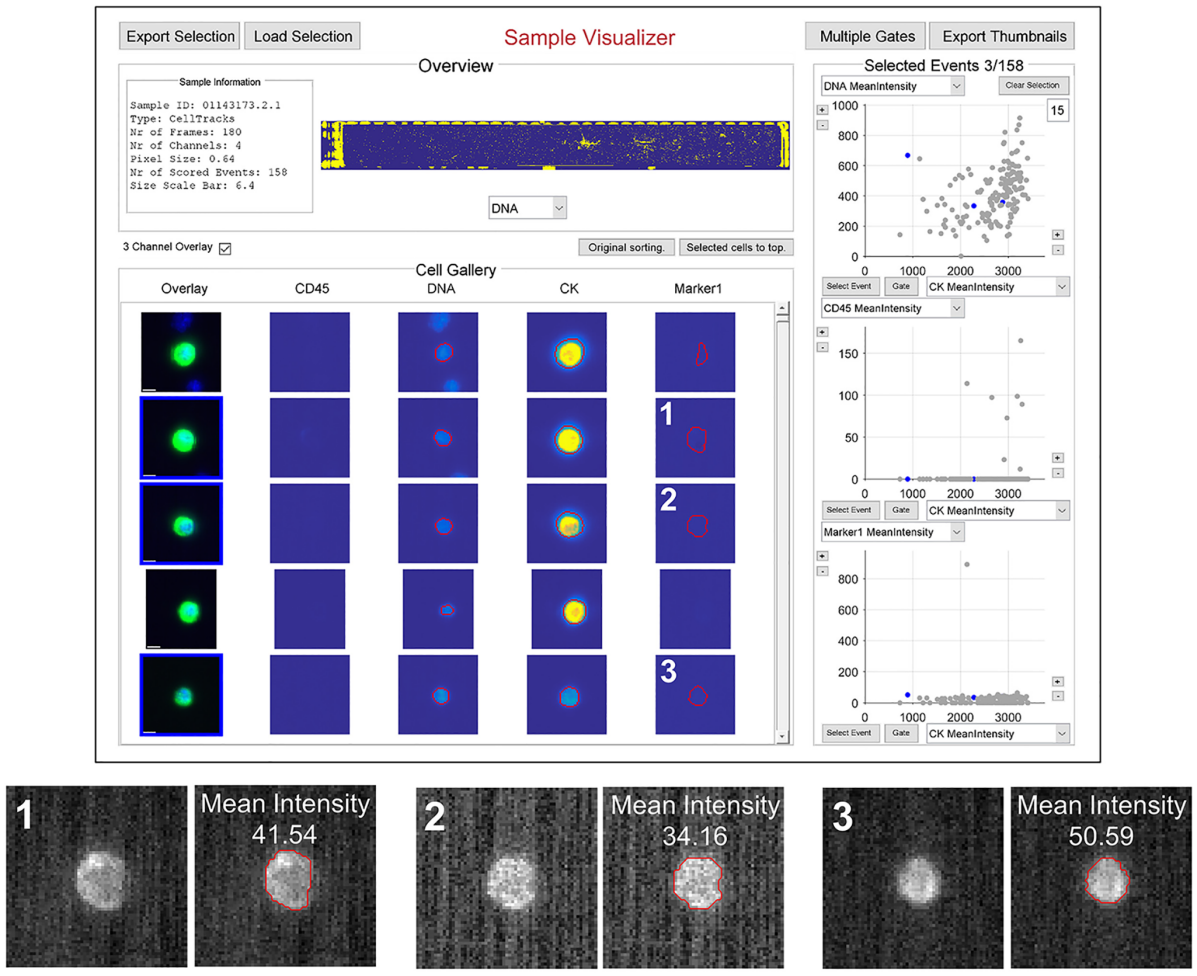
Sample Visualizer of ACCEPT. In the scatter plots 3 of the 158 objects are depicted blue and the corresponding thumbnail images are highlighted. In this example ‘Marker1’ represents signals for HER-2. The corresponding HER-2 images are shown below the Sample Visualizer, in the right image the red line indicates the boundary detected by ACCEPT of the identified CTC and the number indicates the median value of the HER-2 staining within this boundary. Size bar in overlay: 6.4μm.

In Fig 1 the visualization of CTCs in the ACCEPT toolbox is shown. Some sample information is presented on the top left with a scaled overview image of the sample next to it. The main components of the visualization window are the thumbnail gallery on the left and three scatter plots on the right. The thumbnail gallery presents an overlay image of the first three fluorescent markers (CD45, DAPI, CK) and next to it a thumbnail image of every single fluorescent channel. While the overlay image is scaled, the single channels present the full, unscaled intensity range to prevent misinterpretation of the signal. For the three marked cells a scaled version is depicted below the visualization window. While the scaled visualization gives the impression that all three cells have a similar expression, a precise segmentation of the signal as done in ACCEPT (indicated by the red contour) shows that their mean intensity does differ. The standard deviation of the background signal is in this case 6.6 on average. A two-sided t-test shows that the intensity difference between thumbnail one and three as well as between two and three is statistically meaningful. In the unscaled image, interpretation faults like this are prevented. Very dim signals (like the HER-2 expression of the three selected cells) are difficult to see, but the red contour shows that a signal is presented and the dots in the scatter plots on the right visualize the extracted measurements such as the mean intensity. In Fig 1 we plotted the CK versus DAPI mean intensity, CK versus CD45 mean intensity and CK versus HER-2 mean intensity. Moreover, right clicking on an image in the thumbnail gallery opens a scaled visualization.

### HER-2 expression on breast cancer cell lines

**Fig 2:**
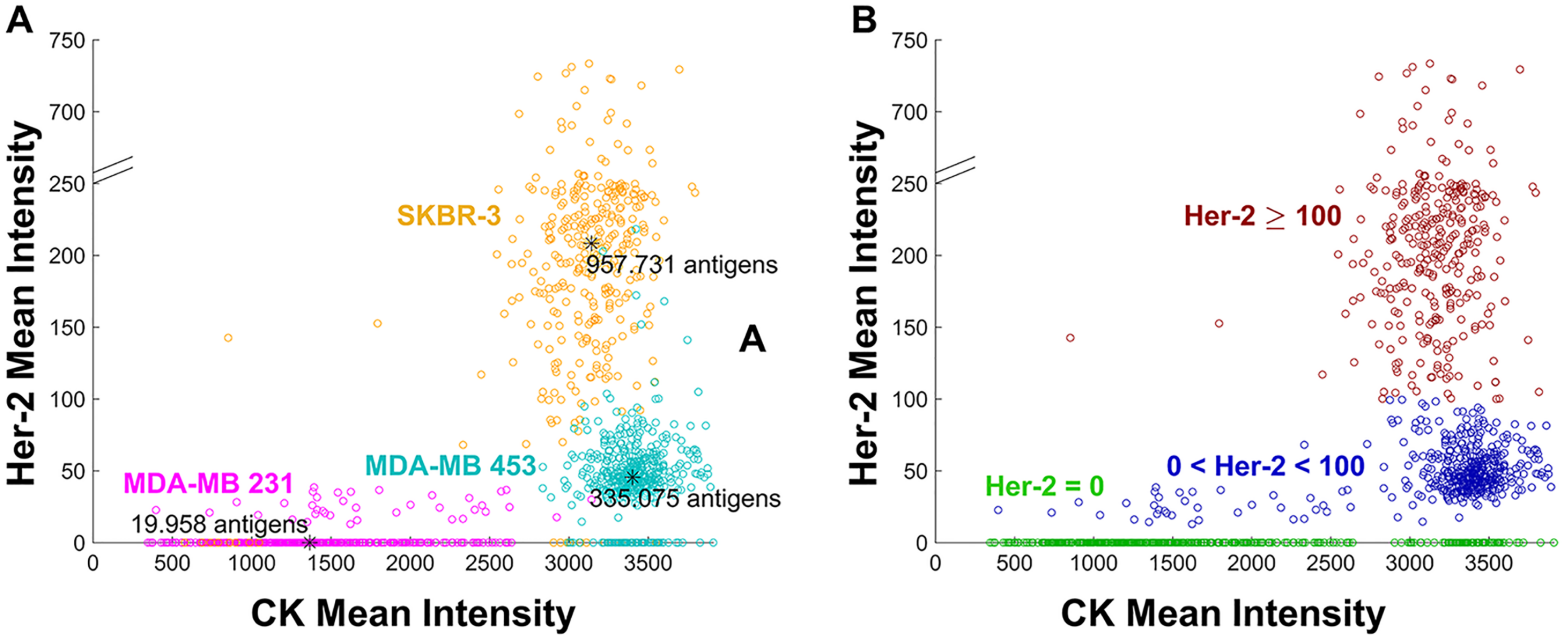
Cytokeratin and HER-2 mean intensities of cells in breast cancer cell lines MDA-MB 231, MDA-MB 453 and SKBR-3. Panel A, 373 MDA-MB 231 cells (magenta), 496 MDA-MB 453 cells (cyan), 361 SKBR-3 cells (orange). Average number of HER-2 antigens included for each cell line. Panel B classification of the MDA-MB 231, MDA-MB 453 and SKBR-3 into 428 negative HER-2 (green), 462 dim HER-2 (blue) and 340 bright HER-2 (red) expressing cells identified by cluster analysis.

To define a threshold for positive HER-2 expression, we evaluated the HER-2 mean intensity of the three breast cancer cell lines SKBR-3 (3+), MDA-MB 453 (2+) and MDA-MB 231 (0 or 1+). Fig 2A depicts the scatter plot of the CK mean intensities versus the HER-2 mean intensities for each cell line. Based on the HER-2 expression shown in panel A we defined 2 thresholds to distinguish between HER-2 negativity (0 or 1+), dim HER-2 expression (2+) and bright HER-2 (3+) expression: a dim HER-2 expression has a mean intensity between 0 and 100 and a bright HER-2 expression has a mean intensity above 100. With these thresholds 87.15% of the cells were correctly classified (Fig 2B). Note that a cutoff of 0 is sufficient to distinguish between HER-2 negative and dim HER-2 expression since a separating gap between these two classes is automatically constructed by our segmentation method. Yet we see that overall the HER-2 expression is very low; the maximal possible intensity is 4095 while the highest measured mean intensity in the 3+ positive cell line is around 1000. To ensure that the measured signal intensity correlates to the number of antigens we compared the median intensity value and coefficient of variation (CV) for each of the three cell lines to the number of HER-2 antigens determined by measuring their mean expression levels by flow cytometer using the BD Quantibrite^™^ Beads PE Fluorescence Quantitation Kit (BD Biosciences, San Jose, CA, USA). The average expression of HER-2 on the SKBR-3 cells was 957.731 (CV 78.4%) HER-2 antigens, MDA-MB 453 had 335.075 (CV 62.9%) HER-2 antigens and MDA-MB 231 expressed 19.958 (CV 108.1%) HER-2 antigens. This is in line with literature [20]. These values result in a linear correlation (see S1 Fig) and show that the measured mean intensity is a valid measure for the HER-2 expression. This was also previously shown in [21]. The authors showed that the HER-2 signal of the cells found using this assay relates to the gene copy number in their cohort.

### HER-2 expression of CTC in metastatic breast cancer patients

**Fig 3:**
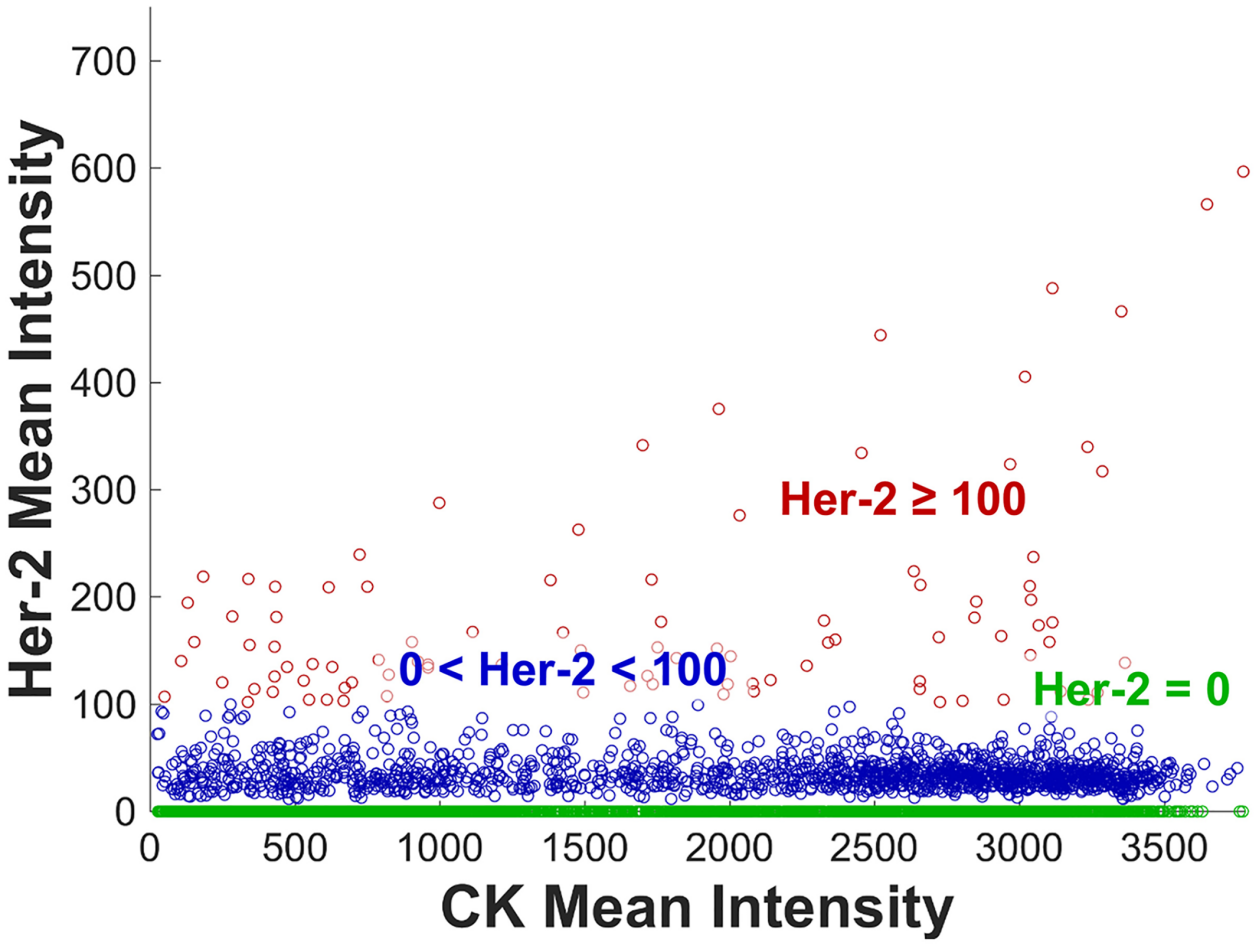
Expression of Cytokeratin and HER-2 on 4084 CTCs in 132 breast cancer patients. HER-2 negative CTCs are colored in green, dim HER-2 (0 < HER-2 < 100) in blue and bright HER-2 (≥100) in red.

We evaluated the HER-2 expression of 4084 CTCs from 132 metastatic breast cancer patients and applied the same thresholds as with the cell lines in Fig 2. Fig 3 shows a scatter plot with the CK mean intensity (x-axis) versus the HER-2 mean intensity (y-axis). Again, all CTCs not expressing HER-2 are depicted in green (61.4%), CTCs with a dim HER-2 expression between 1 and 99 are labeled blue (36.2%) and those with a bright HER-2 mean expression larger or equal to 100 are colored in red (2.4% of all CTCs).

### Heterogeneity of HER-2 expression per patient

**Fig 4:**
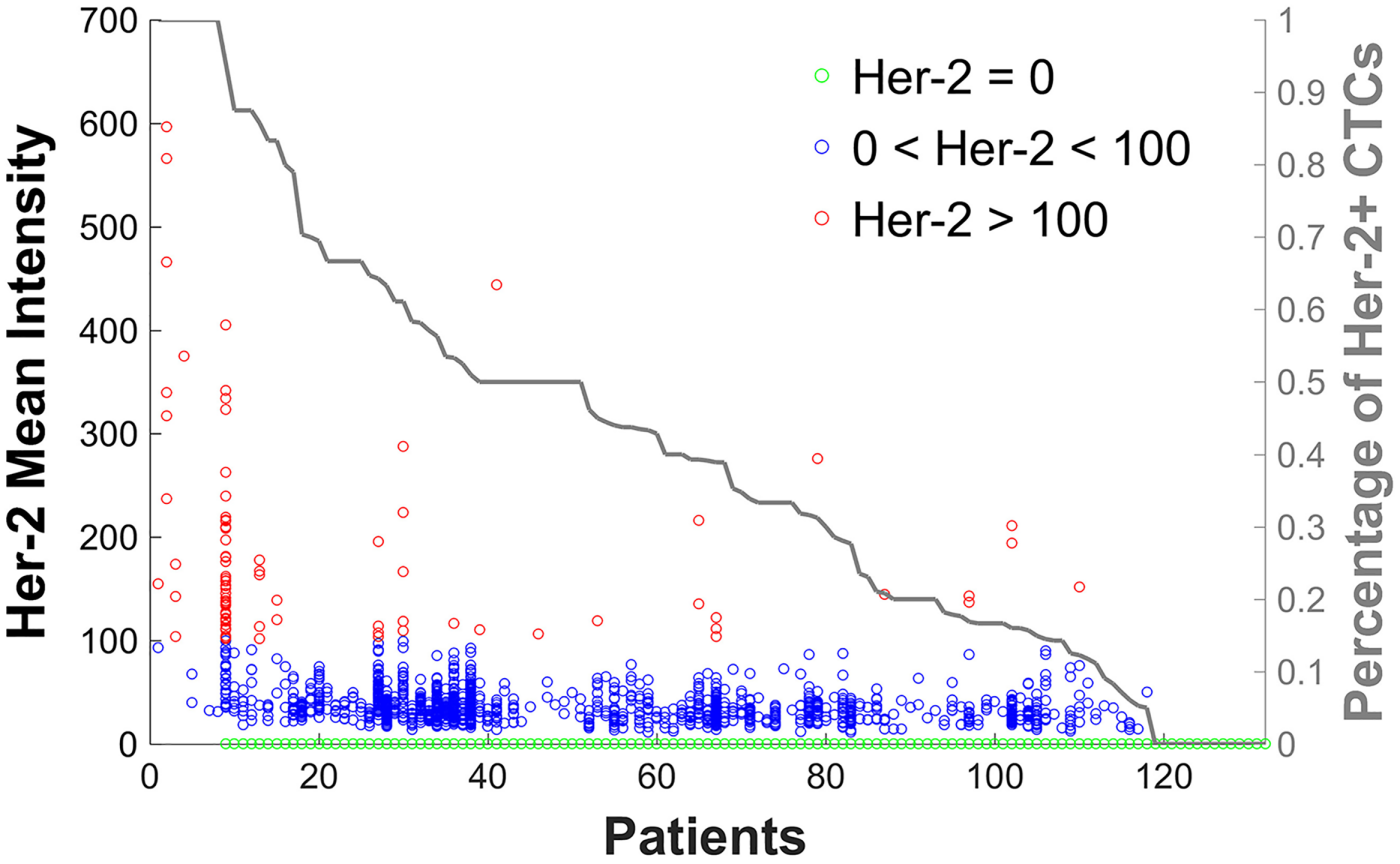
HER-2 expression in CTCs in each of the 132 patients. Patients are sorted according to the percentage of HER-2+ CTCs using a threshold of (>0). HER-2 negative CTCs are colored in green, dim HER-2 in blue (0 < HER-2 < 100) and bright HER-2 (≥100) CTCs red.

For each of the 132 patients we evaluated the heterogeneity of HER-2 expression in CTCs. The results are shown in Fig 4 and are sorted from left to right according to decreasing percentages of CTCs that are HER-2 dim or bright (HER-2 > 0). Again, HER-2 negative CTCs are colored in green, dim HER-2 in blue and bright HER-2 in red. In 8 (6.1%) patients all CTCs expressed HER-2, in 14 (10.6%) patients none of the CTCs expressed HER-2 and in 110 (83.3%) patients various HER-2 expression levels on CTCs were observed. Thus, for most patients not all CTCs express or lack HER-2, most patients rather present CTCs with varying levels of HER-2 signal.

### HER-2 expression of CTC assessed by operators and ACCEPT

**Fig 5:**
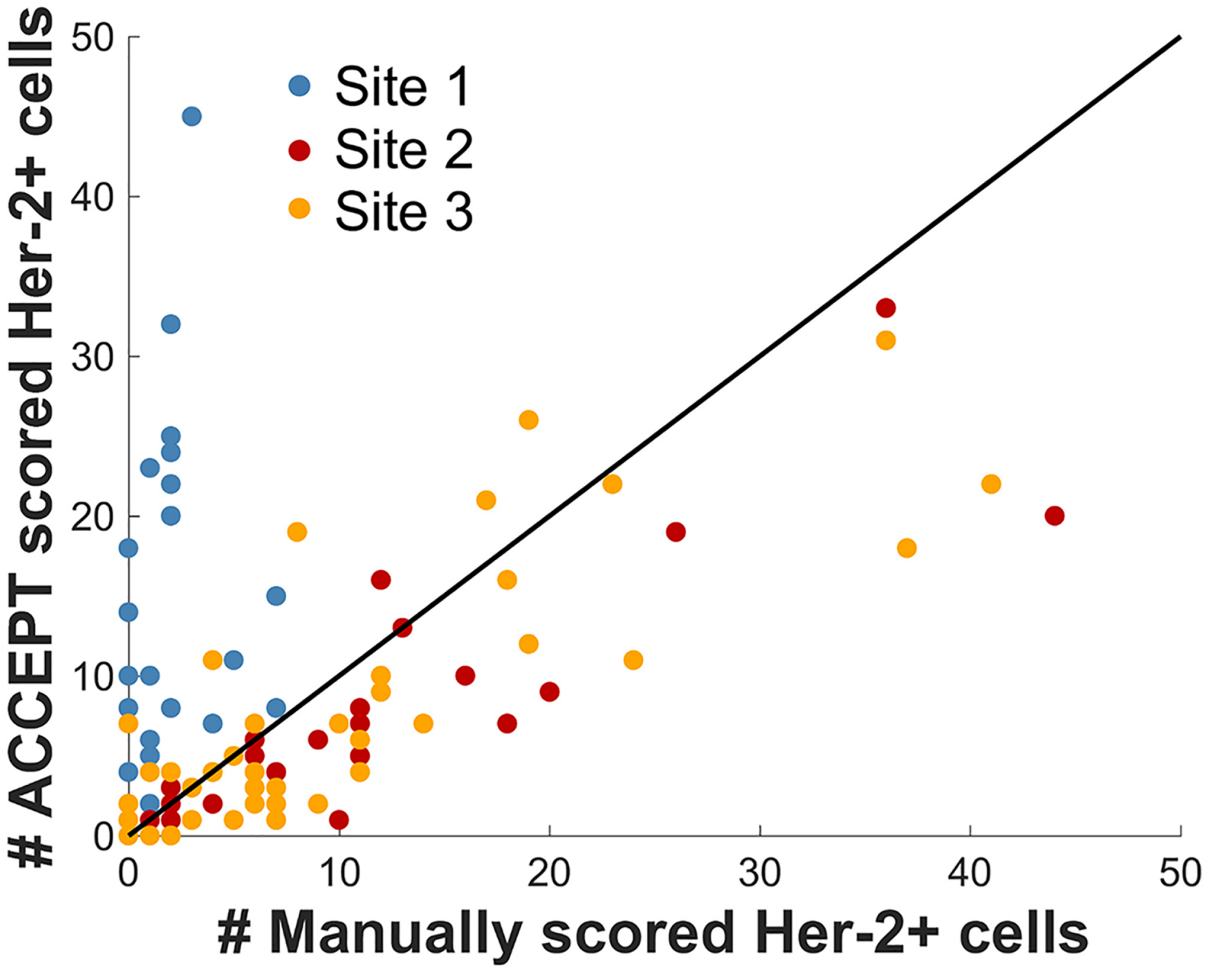
Number of manually scored HER-2+ cells versus number of automatically scored HER-2+ cells using a threshold of >0. Samples of 132 patients were investigated.

To assess whether the defined thresholds correlate with manual scoring, we compared for each of the 132 samples the number of automatically scored HER-2 positive CTCs (HER-2 mean intensity > 0) with the number of CTCs that were manually scored as HER-2 positive (2+ or 3+) by the three clinical sites. The results are shown in Fig 5. We observe a good agreement between the scores of sites 2 and 3, yet on average they manually scored more cells as HER-2 positive than our automated procedure. Nevertheless, there is a reasonably high Pearson correlation between the manual and automatic scores for site 2 (coefficient r = 0.82) and site 3 (coefficient r = 0.98). Operators of site 1 were much stricter with their definition of HER-2 positivity and scored fewer cells as positive compared to the automated approach. This results in a much lower correlation coefficient (r = 0.51). To compare the number of manually and automatically scored cells to a “ground truth” solution, further experiments are necessary to obtain images of cells where the HER-2 expression is known. Yet, in this work, we concentrate on retrospectively studying samples that were investigated before. The main goal of the Marker Characterization tool of the ACCEPT toolbox is to reproducibly quantify manually scored cells and aid users to unify their scoring results. Yet, any approach that is still to some part manual will not give fully reproducible and unified results. This highly motivates the use of a fully automated approach, as it will be possible in another tool of the ACCEPT software, especially in multi-center studies to unify the scoring results.

### Influence of quantitative display of HER-2 expression

In Fig 1 we have shown the difference between the traditionally scored visualization of fluorescent intensity (three examples at the bottom) and the visualization we used in the ACCEPT tool. Since it is difficult to evaluate the intensity of a fluorescent signal based on a scaled image, this way of visualizing data could be a major cause for the high inter-user variability observed in manual scores. Therefore, we developed a tool to allow the operators to rate the HER-2 expression with and without the quantitative display of HER-2 expression in the form of a scatterplot.

**Fig 6:**
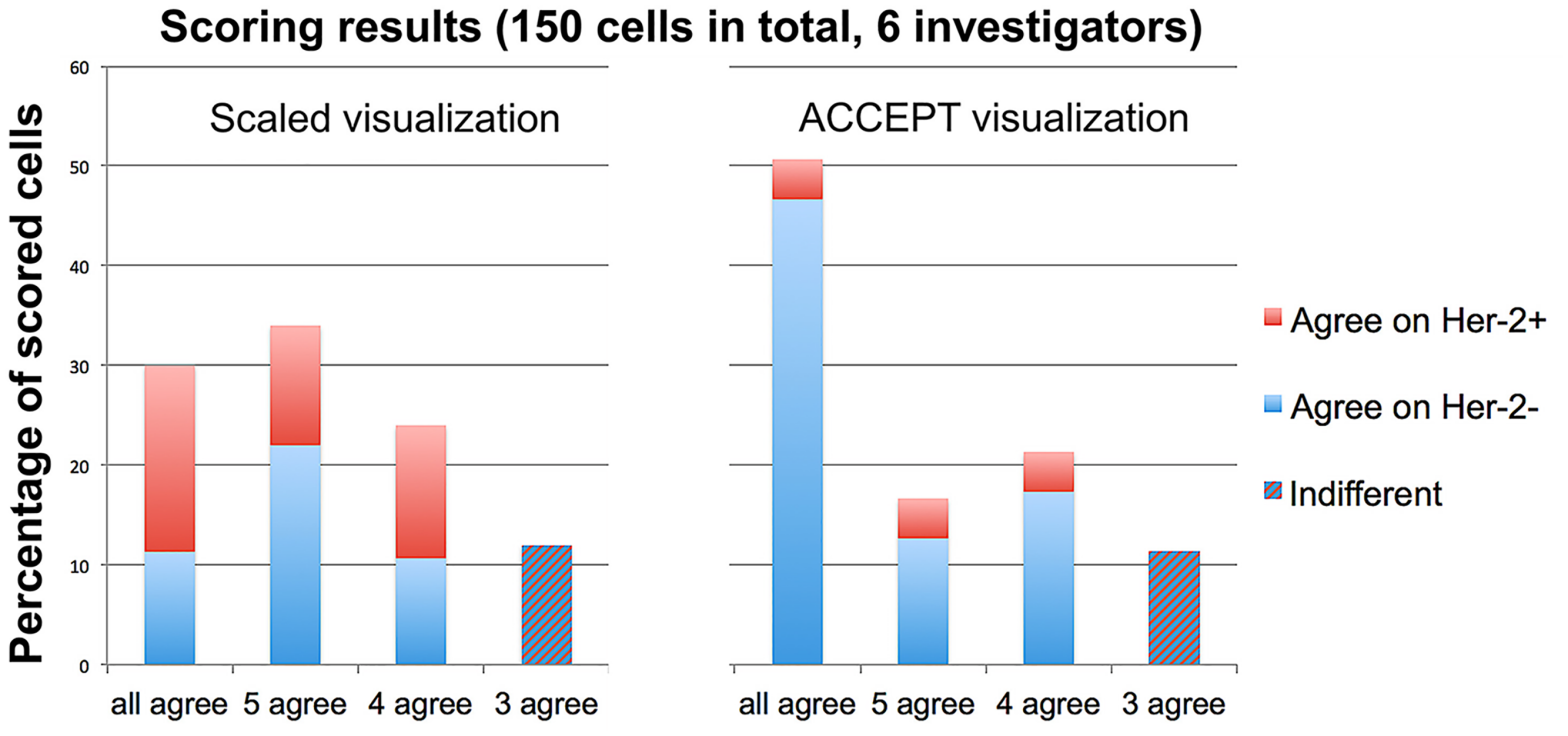
Comparisons of HER-2 assessment by different sites using ACCEPT versus CellTracks Analyzer II^®^ (Menarini Silicon Biosystems Inc) visualization. Indicated are the percentages of scored CTCs where all, five, four or only three out of six investigators agreed on the HER-2 status. In the case of 3 agree indifferent means that three investigators vote for HER-2 positive and the other three for HER-2 negative.

To test if the concordance between different operators can be increased when our tool is used for scoring, a total of 150 randomly chosen images of CTCs were sent to six different investigators for scoring HER-2 positivity. The results are shown in Fig 6. For scaled visualization images in 30 percent of the cases all investigators agreed with a major part of agreement on positivity (red box) although a large part of the cells had nearly no HER-2 expression by quantitative assessment of HER-2 mean intensities. With the quantitative display in ACCEPT this percentage of full agreement can be increased to over 50 percent (right graph). Here the investigators agreed mostly on HER-2 negativity (blue box). Thus, the scaled visualization has a high sensitivity but also a lot disagreement while the quantitative display leads to more true negatives and therefore a higher specificity and less disagreement. To investigate which cells have the highest probability to result in disagreement of reviewers, we compared the Cytokeratin and HER-2 mean intensity of each of the 150 cells to the number of reviewers that agree on their HER-2 status. The results for both, the scaled and the ACCEPT visualization, are shown in the supplemental figure S2 Fig. We see that the quantitative display significantly decreases the spread of user disagreement. Yet, the Marker Characterization tool can still only help users to make their decision therefore there are still 49% where at least one reviewer has a different opinion than the other ones. This illustrates the need for either automated classification of CTCs as HER-2 positive CTCs or classification of HER-2 positive CTCs by using the ACCEPT quantitative displays.

## Discussion

Since treatment options for cancer patients are increasing rapidly and concurrently there is a growing demand for biomarkers that can predict the most effective therapy. Availability of tumor material before the start of a new line of therapy is imperative to determine the presence of treatment targets to eradicate the tumor. However, a tumor or its metastasis might not always be accessible for biopsy. Therefore, isolation of tumor cells from blood represents a unique opportunity to obtain tumor material providing its latest genotypic and phenotypic fingerprint. After demonstration that tumor cells can be reproducibly isolated from blood and that their presence is related to poor clinical outcome [22] many new technologies have been introduced to detect and isolate CTCs [13,14,15,23]. The lack of an automated unified approach to designate a cell as a CTC and determine what is and what is not expressed is however impeding progress of the field. To address this issue, an open source image analysis program ACCEPT is being developed in a consorted European effort enabling comparisons between platforms and providing accurate and reproducible information. Here we introduce the first applications of ACCEPT and demonstrate the ability to extract relative expression of antigens expressed by CTCs. In addition, we show that the use of scatterplots and expression levels of the antigens next to the fluorescence images helps to reduce inter reader variability in terms of scoring of HER-2 positivity. Increased concordance in the expression of targetable biomarkers will optimize the performance and results of clinical multicenter studies such as the DETECT trial [24].

Of particular interest in this study is the large heterogeneity observed in the HER-2 expression of CTCs in breast cancer patients. After evaluation of the HER-2 signal CTCs were divided up into three groups: HER-2 negative, HER-2 low and HER-2 high. This division was based on the HER-2 expression levels measured on MDA-MB 231, MDA-MB 453 and SKBR3 breast cancer cells. A more meaningful division may be to discriminate subgroups based on the effect of a HER-2- targeted treatment on the CTCs directly. This could for example be achieved by measuring HER-2 expression levels of CTCs before and after administration of a HER-2 targeted therapy.

In earlier studies, we developed computer generated CTC definitions using overall survival of the patients as the training parameter [21] and extract information from the identified CTC [25,26]. In this study, we used advanced mathematical approaches to identify objects in the images and to extract features from the identified objects [16]. More importantly, we make the program available for all researchers with an interest in identifying and characterizing CTCs or other objects and enable the comparison of fluorescence signals generated with different imaging platforms. By using the tools provided in ACCEPT differences in the effectiveness of different treatments targeting the HER-2 receptor can be assessed. The ACCEPT program and manual can be downloaded from https://github.com/LeonieZ/ACCEPT.

Besides a more reliable and reproducible quantification of therapeutic marker expression, our ultimate goal is to develop a common definition of a CTC. The large variety of cancer and phenotypes makes it of course very challenging but using the input of a variety of users applying a variety of platforms together with the current breakthroughs in the field of machine learning and imaging may help to understand differences and similarities in the CTCs between different cancer and phenotypes in the future and enables us to find a common CTC definition spanning at least a wide range of cancer and phenotypes.

Until then, a unified approach to the analysis of fluorescent images across CTC isolation platforms and CTC phenotypes may increase robustness and lead to a higher reproducibility of results as we have seen it in this study. In its current version, the software can load images from several CTC isolation platforms. Moreover, it contains a general tiff-loader if no specific sample information should be loaded. Loaders for different microscope system can be easily integrated and are work in progress. Further features and tools will be available throughout the IMI CANCER-ID program.

## Supporting information

S1 Fig Linear calibration of the number of HER-2 antigens and the measured HER-2 signal intensity. Values plotted for each of the investigated cell lines together with the corresponding line equation and regression value.
(TIF)

S2 Fig Comparisons of HER-2 assessment using ACCEPT versus CellTracks Analyzer II^®^ (Menarini Silicon Biosystems Inc) visualization in relation to measured mean intensities. Expression of Cytokeratin and HER-2 on the 150 randomly chosen images of CTCs that were sent to six different investigators for scoring HER-2 positivity. Marker colors indicate if all, five, four or only three out of six investigators agreed on the HER-2 status. Panel A and C correspond to the scaled visualization (C is a zoom-in of A) and panel B and D correspond to the ACCEPT visualization (D is a zoom-in of B).
(TIF)

S1 Table Data tables. (XLSX)
