# Supplementary figures and images for: Quantifying HER-2 expression on circulating tumor cells by ACCEPT
Source: PLoS One. 2017 Oct 30;12(10):e0186562. doi: 10.1371/journal.pone.0186562 (PMC5662084; doi:10.1371/journal.pone.0186562)

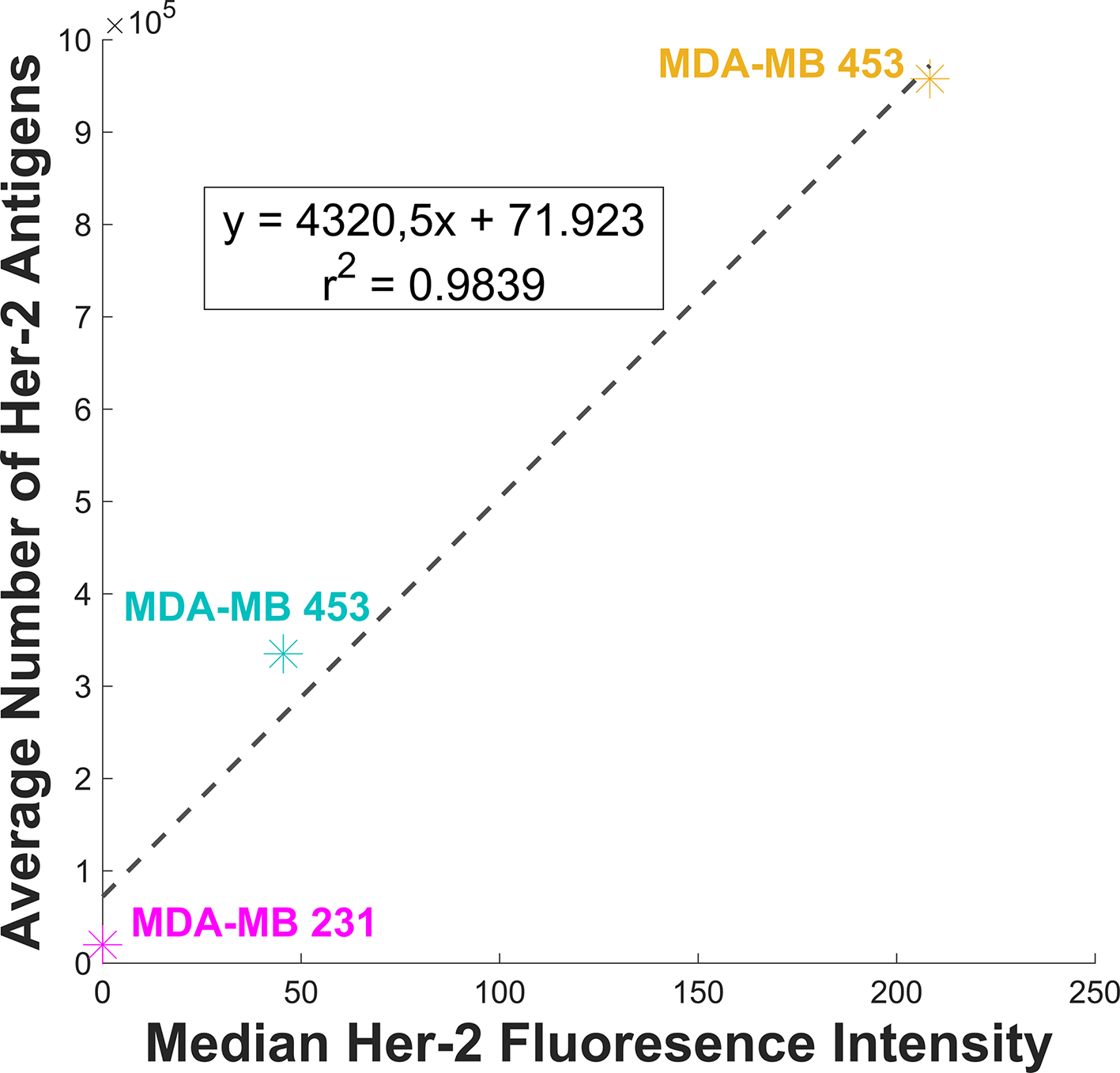

Supplement: S1 Fig — Values plotted for each of the investigated cell lines together with the corresponding line equation and regression value. (TIF) [file pone.0186562.s001.tif]

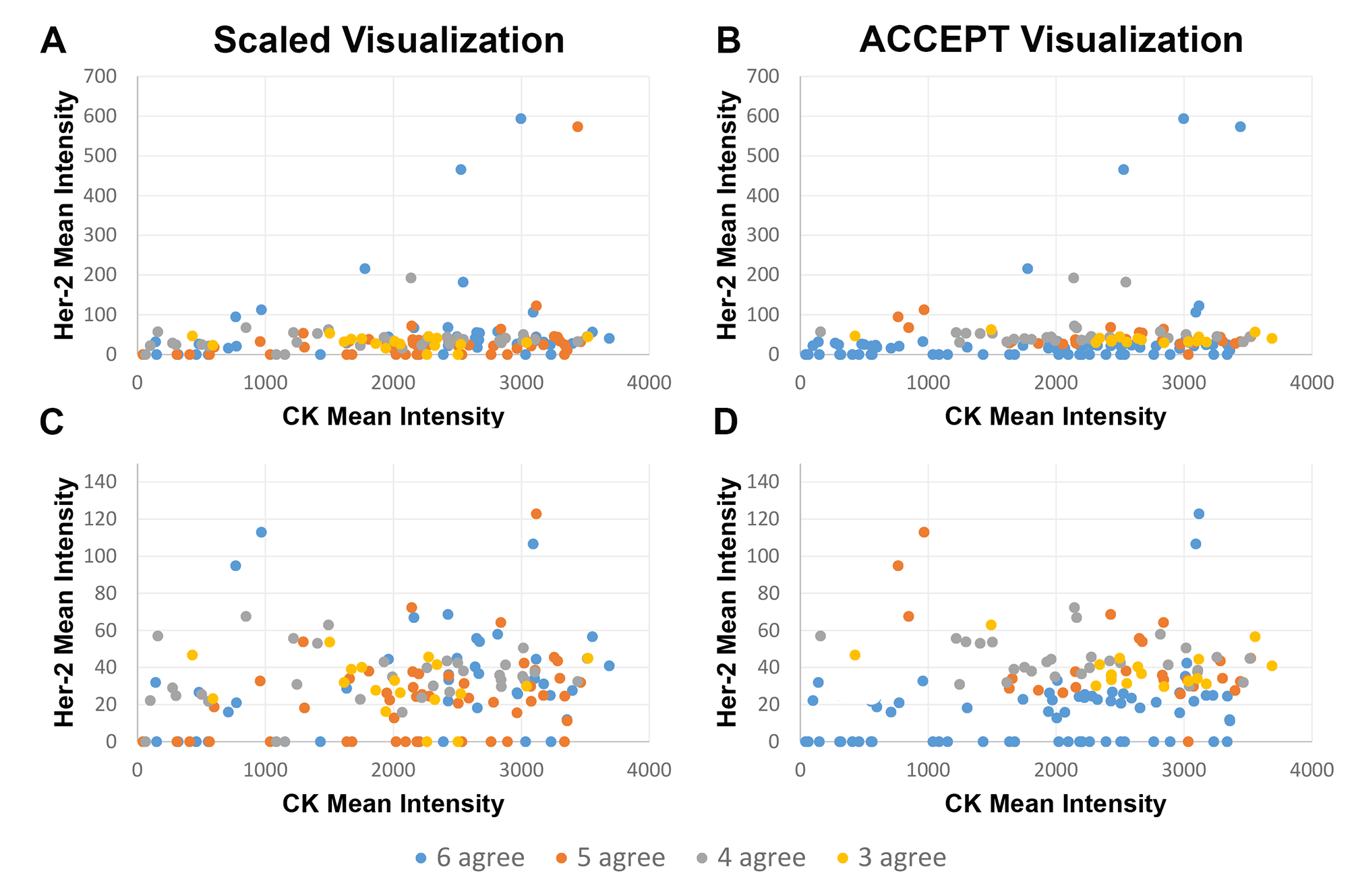

Supplement: S2 Fig — Expression of Cytokeratin and HER-2 on the 150 randomly chosen images of CTCs that were sent to six different investigators for scoring HER-2 positivity. Marker colors indicate if all, five, four or only three out of six investigators agreed on the HER-2 status. Panel A and C correspond to the scaled visualization (C is a zoom-in of A) and panel B and D correspond to the ACCEPT visualization (D is a zoom-in of B). (TIF) [file pone.0186562.s002.tif]
